# Growth mechanisms of MgO nanocrystals *via* a sol-gel synthesis using different complexing agents

**DOI:** 10.1186/1556-276X-9-134

**Published:** 2014-03-21

**Authors:** Mohd Sufri Mastuli, Norlida Kamarulzaman, Mohd Azizi Nawawi, Annie Maria Mahat, Roshidah Rusdi, Norashikin Kamarudin

**Affiliations:** 1 School of Chemistry and Environmental Studies, Faculty of Applied Sciences, Universiti Teknologi MARA, Shah Alam, Selangor 40450, Malaysia; 2 School of Physics and Materials Studies, Faculty of Applied Sciences, Universiti Teknologi MARA, Shah Alam, Selangor 40450, Malaysia; 3 Centre for Nanomaterials Research, Institute of Science, Universiti Teknologi MARA, Level 3, Block C, Shah Alam, Selangor 40450, Malaysia

**Keywords:** MgO, Nanostructured materials, Crystal growth, Sol-gel process, Complexing agent

## Abstract

In the preparation of nanostructured materials, it is important to optimize synthesis parameters in order to obtain the desired material. This work investigates the role of complexing agents, oxalic acid and tartaric acid, in the production of MgO nanocrystals. Results from simultaneous thermogravimetric analysis (STA) show that the two different synthesis routes yield precursors with different thermal profiles. It is found that the thermal profiles of the precursors can reveal the effects of crystal growth during thermal annealing. X-ray diffraction confirms that the final products are pure, single phase and of cubic shape. It is also found that complexing agents can affect the rate of crystal growth. The structures of the oxalic acid and tartaric acid as well as the complexation sites play very important roles in the formation of the nanocrystals. The complexing agents influence the rate of growth which affects the final crystallite size of the materials. Surprisingly, it is also found that oxalic acid and tartaric acid act as surfactants inhibiting crystal growth even at a high temperature of 950°C and a long annealing time of 36 h. The crystallite formation routes are proposed to be via linear and branched polymer networks due to the different structures of the complexing agents.

## Background

Magnesium oxide (MgO) is a versatile metal oxide having numerous applications in many fields. It has been used as a catalyst and catalyst support for various organic reactions [1,2], as an adsorbent for removing dyes and heavy metals from wastewater [3,4], as an antimicrobial material [5], as an electrochemical biosensor [6] and many other applications. Conventionally, MgO is obtained via thermal decomposition of various magnesium salts [7-9]. The drawback with this method of obtaining MgO is the large crystallite size with low surface area-to-volume ratio that limits its applications for nanotechnology. Some properties of MgO, such as catalytic behaviour, can be further improved if it is used as nanosized particles compared to micron-sized particles. Therefore, the formation of MgO nanostructures with a small crystallite size of less than 100 nm and homogeneous morphology has attracted much attention due to their unique physicochemical properties including high surface area-to-volume ratio. It is widely accepted that the properties of MgO nanostructures depend strongly on the synthesis methods and the processing conditions. Much effort has been devoted to synthesize MgO nanostructures using various methods such as precipitation [10], solvothermal [11], chemical vapour deposition [12], electrochemical [13], sonochemical [14], microwave [15], electron spinning [16], combustion [17], template [18] and carbothermic reduction [19]. Each method has its own advantages and disadvantages. An important issue regarding synthesis and preparation of nanostructured MgO is controlling the parameters in order to obtain a more uniform size as well as morphology of the nanoparticles.

Over the past decades, various starting materials were used in the synthesis methods producing nanosized MgO that may give multiple morphologies. Precursors that may be obtained from the synthesis methods may take many forms such as magnesium hydroxide [10,15], magnesium carbonate [20,21] and basic magnesium carbonate [22,23]. Each precursor is annealed at a different temperature to produce highly crystalline and pure MgO. Another precursor, magnesium oxalate dihydrate (MgC_2_O_4_ · 2H_2_O), has also received considerable interest among researchers [24,25]. A sol-gel method is a promising technique for the formation of magnesium oxalate dihydrate followed by annealing at a suitable temperature to form MgO. The advantages are its simplicity, cost-effectiveness, low reaction temperature, high surface area-to-volume ratio, narrow particle size distribution and high purity of the final product. Early attempts to prepare magnesium oxalate dihydrate were by using either magnesium methoxide or magnesium ethoxide that was reacted with oxalic acid in ethanol to form a precursor based on the sol-gel reaction [26-28]. Later on, inorganic salts like magnesium nitrate hexahydrate [29-31], magnesium chloride hexahydrate [32] and magnesium acetate tetrahydrate [33] are preferred. The sol-gel reaction of magnesium oxalate dihydrate and annealing of the obtained precursors give various morphologies of MgO nanostructures [29-32]. However, the controlled synthesis of MgO nanostructures with homogeneous morphology, small crystallite size and narrow size distribution is a challenging aspect to be investigated. Understanding the growth mechanism is an important part of controlling the size of nanostructures. The synthetic strategies of tailoring the size and shape of the nanostructures are key issues to be addressed in nanomaterials research.

To the best of our knowledge, there is no report on the effect of the molecular structure of complexing agents on MgO nanostructures even though the control of nanostructures presents an important part of nanotechnology work. Our work is focused on the effect of complexing agents on the MgO nanostructures finally obtained after synthesis. The study is done by using two different types of complexing agents, namely oxalic acid and tartaric acid. The molecular structures of these complexing agents are taken into account, and chemical reactions involving the complexing agents and site attachments of the Mg^2+^ and O^2−^ ions in the process of the formation of MgO nanostructures are considered. Results give some insights into the mechanisms of size and shape formation of MgO nanostructures.

## Methods

All the chemicals used were analytical grade and directly used as received without further purification. Magnesium acetate tetrahydrate, Mg(CH_3_COO)_2_ · 4H_2_O (Merck, 99.5% purity); oxalic acid dihydrate, C_2_H_2_O_4_ · 2H_2_O (Merck, >98% purity); tartaric acid, C_4_H_6_O_6_ (Merck, 99.5% purity); and absolute ethanol, C_2_H_5_OH (J. Kollin Chemical, 99.9% purity) were used for the formation of MgO nanostructures. These chemicals were manufactured by Merck KGaA Company at Darmstadt, Germany. The MgO samples were synthesized using the sol-gel method with two different types of complexing agents, namely oxalic acid and tartaric acid. Magnesium acetate tetrahydrate of mass 53.2075 g was initially dissolved in 150 ml of absolute ethanol under constant stirring. The pH of the solution was then adjusted to pH 5 using 1 M oxalic acid. The mixture was continuously stirred until a thick white gel was formed. The gel formed was left overnight for further gelation process before being dried in an oven at 100°C for 24 h. The dried materials were grounded using mortar and pestle to produce fine powder precursors. Subsequently, the precursors were annealed at 950°C for 36 h to form MgO nanostructures. The samples were identified as MgO-OA and MgO-TA for complexing agents oxalic acid and tartaric acid, respectively.

All the MgO samples were systematically characterized using various instruments. The thermal profiles of the precursors were studied using simultaneous thermogravimetric analysis (STA; SETARAM SETSYS Evolution 1750, Caluire, France). This thermal analysis method has the advantage of giving very accurate calorimetric data that is simultaneously measured and calculated with weight loss. It gives more accurate insight into the processes occurring while the precursor is heated. The obtained precursors were heated from room temperature to 800°C at a heating rate of 10°C min^−1^. The X-ray diffraction (XRD) patterns of MgO-OA and MgO-TA were obtained by XRD PANalytical X'Pert Pro MPD (Almelo, Netherlands) with CuK_α_ radiation. The Bragg-Brentano optical configuration was used during the data collection. The size and morphology of the MgO crystallites were determined using a field emission scanning electron microscope (FESEM; JEOL JSM-7600 F, Tokyo, Japan) and a transmission electron microscope (TEM; JEOL JEM-2100 F, Tokyo, Japan).

## Results and discussions

**Figure 1:**
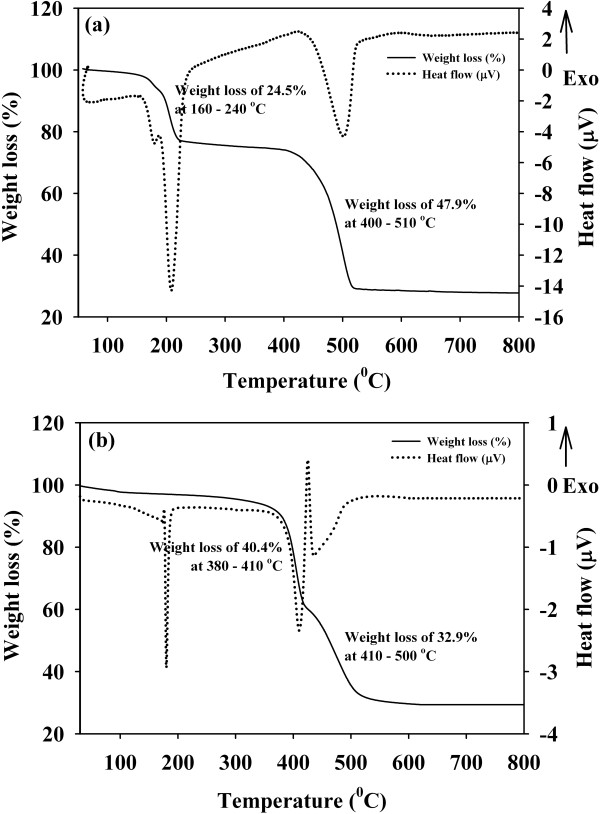
**TG/DSC curves of the precursors. (a)** Magnesium oxalate dihydrate and **(b)** magnesium tartrate, as a precursor for MgO-OA and MgO-TA, respectively.

In this sol-gel method, the metal salt (magnesium acetate tetrahydrate) and the complexing agents (oxalic acid and tartaric acid) were dissolved in ethanol to form a mixture of cation (Mg^2+^) and anion (C_2_O_4_^2−^ or C_4_H_4_O_6_^2−^). At pH 5, it is believed that the complexation and polymerization processes took place simultaneously resulting in the formation of a thick white gel which is dried and a white precursor is obtained. Chemical reactions (1) and (2) show the formation of the precursors, hydrated MgC_2_O_4_ and anhydrous MgC_4_H_4_O_6_, for the oxalic acid and tartaric acid routes, respectively. Acetic acid and water as side products of the sol-gel route were evaporated during the drying process for the formation of precursors. Even though the boiling point of acetic acid is 119°C, the process of evaporation occurs at lower temperatures as well and must have evaporated during the long drying process at 100°C. Thus, this process did not appear in the thermal profiles of the precursors at 119°C as shown in Figure 1a,b. A small and very gradual weight loss can be observed at about ambient to about 160°C for both precursors that correspond to the removal of water still remaining in the precursors.

(1) $$\begin{array}{ll} \mathrm{Mg}{\left({\mathrm{CH}}_{3}\mathrm{COO}\right)}_{2}\cdot 4H_{2}O & +H_{2}C_{2}O_{4}\cdot 2H_{2}O\to \underset{\left(\mathrm{MgO}‒\mathrm{OA}\;\mathrm{precursor}\right)}{{\mathrm{MgC}}_{2}O_{4}\cdot 2H_{2}O}\\ & +2{\mathrm{CH}}_{3}\mathrm{COOH}+4H_{2}O \end{array}$$

(2) $$\begin{array}{ll} \mathrm{Mg}{\left({\mathrm{CH}}_{3}\mathrm{COO}\right)}_{2}\cdot 4H_{2}O & +H_{6}C_{4}O_{6}\to \underset{\left(\mathrm{MgO}‒\mathrm{TA}\;\mathrm{precursor}\right)}{{\mathrm{MgC}}_{4}H_{4}O_{6}}\\ & +2{\mathrm{CH}}_{3}\mathrm{COOH}+4H_{2}O \end{array}$$

Figure 1a shows the thermal profile of the MgO-OA precursor. It exhibits two major weight losses which are ascribed to the dehydration and decomposition of the precursor. The first weight loss occurred in the temperature range of 160°C to 240°C accompanied by two endothermic peaks at about 180°C and 210°C. The first endothermic peak is due to the removal of water, and the second endothermic peak is attributed to the dehydration of MgC_2_O_4_ · 2H_2_O. This weight loss is 24.5% which agrees very well with the proposed weight loss in chemical reaction (3). However, no corresponding weight loss is observed for the MgO-TA precursor as can be seen from Figure 1b. It is then clear that the routes of MgO formation from these two synthesis methods are different. For the MgO-TA precursor (Figure 1b), the sharp endothermic peak at about 190°C is due to an isomorphic transformation of phase without change in mass as similarly observed by several researchers before [34-36]. The second weight loss of the MgO-OA precursor of about 47.9% between 400°C and 510°C is attributed to the decomposition of MgC_2_O_4_ to MgO. A broad endothermic peak at about 500°C is evidence of the reaction occurring resulting in the formation of MgO nanostructures. The weight loss for the formation of MgO-OA is calculated as shown in chemical reaction (4) and found to be 48.5% which is very close to the experimental value of 47.9%. The whole reaction mechanisms are shown below.

(3) $${\mathrm{MgC}}_{2}O_{4}\cdot 2H_{2}O\;\overset{24.3\;\%\;\mathrm{wt}.\;\mathrm{loss}}{\to }\;{\mathrm{MgC}}_{2}O_{4}+2H_{2}O$$

(4) $${\mathrm{MgC}}_{2}O_{4}\overset{48.5\%\;\mathrm{wt}.\mathrm{loss}}{\to }\underset{\left(\mathrm{MgO}‒\mathrm{OA}\right)}{\mathrm{MgO}}\;+\;\mathrm{CO}\;+\;{\mathrm{CO}}_{2}$$

**Figure 2:**
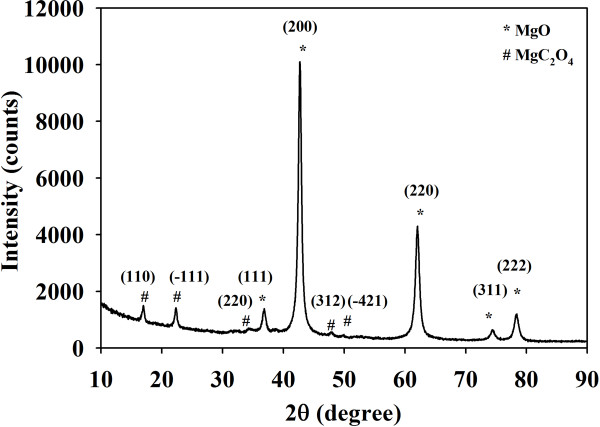
**XRD patterns of the intermediate products.** They are formed when MgC_4_H_4_O_6_ is annealed at 400°C for 30 min.

Thermal gravimetric analysis (TGA) curve of the MgO-TA precursor shows two pronounced weight losses as shown in Figure 1b. The first weight loss occurs at 380°C to 410°C which is 40.4% corresponding to the removal of the two additional carbons within MgC_4_H_4_O_6_. This reaction started with the absorption of heat, but the decomposition is accompanied by the release of heat energy as can be observed by the endothermic and exothermic peaks at 400°C and 430°C respectively shown in the DSC curve. A mixture of MgC_2_O_4_ and MgO is believed to have been formed at this point. To confirm this, the MgO-TA precursor is heated at 400°C for 30 min and the obtained products examined by XRD. Figure 2 shows the XRD pattern of the material, and the phases MgC_2_O_4_ (ICDD reference number 00-026-1222) and MgO (ICDD reference number 01-0178-0430) are confirmed to exist in the sample as indexed in the dataset shown. This validates the proposed chemical reaction as can be seen in Equation 5. The second weight loss of 32.9% occurring at a starting temperature of 410°C to 500°C accompanied by a broad endothermic peak approximately at 450°C can be ascribed to the decomposition of the intermediate product, MgC_2_O_4_ to MgO. These weight losses are in good agreement with the calculated values proposed in the chemical reactions (5) and (6). The whole reaction mechanisms are shown below.

(5) $$\begin{array}{l} {\mathrm{MgC}}_{4}H_{4}O_{6}\overset{39.0\%\;\mathrm{wt}.\mathrm{loss}}{\to }0.9{\mathrm{MgC}}_{2}O_{4}\\ \;+0.1\mathrm{MgO}+2.2\mathrm{CO}+2H_{2}+0.05O_{2} \end{array}$$

(6) $$\begin{array}{l} 0.9{\mathrm{MgC}}_{2}O_{4}\\ \;+0.1\mathrm{MgO}\overset{37.6\%\;\mathrm{wt}.\mathrm{loss}}{\to }\underset{\left(\mathrm{MgO}‒\mathrm{TA}\right)}{\mathrm{MgO}}+\mathrm{CO}\\ \;+0.8{\mathrm{CO}}_{2}+0.05O_{2} \end{array}$$

**Figure 3:**
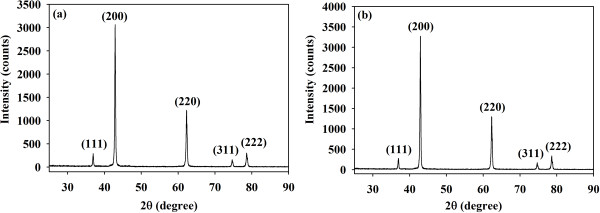
**XRD patterns of the MgO samples.** They are prepared using **(a)** oxalic acid and **(b)** tartaric acid, as a complexing agent.

For both MgO-OA and MgO-TA precursors, the TGs show a horizontal line after 500°C indicating that the MgO stable phase is formed at this temperature. These are confirmed by the XRD results shown in Figure 3. The XRD patterns for both samples are indexed according to ICDD reference number 01-0178-0430 showing a MgO cubic crystal structure of space group *Fm-3 m*. All the fingerprint peaks (111), (200), (220), (311) and (222) are clearly observable. The samples are pure and single phase with no impurities present.

**Figure 4:**
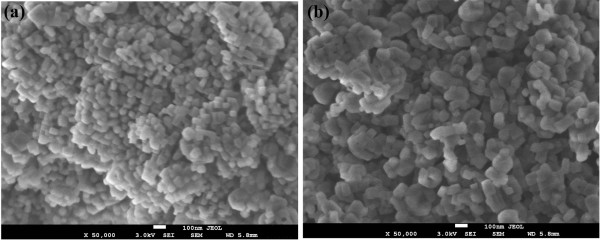
**FESEM micrographs of the MgO samples. (a)** MgO-OA and **(b)** MgO-TA.

**Figure 5:**
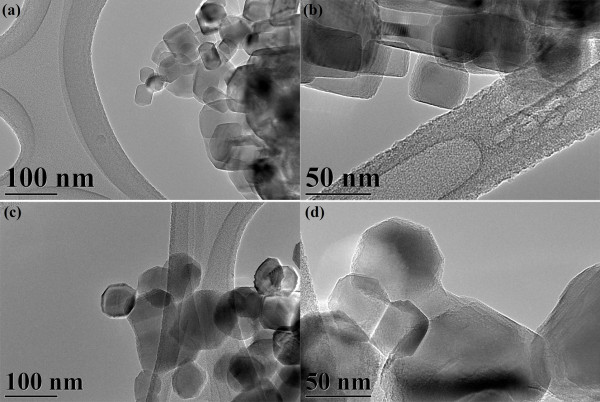
**TEM micrographs of the MgO samples. (a, b)** MgO-OA and **(c, d)** MgO-TA.

**Figure 6:**
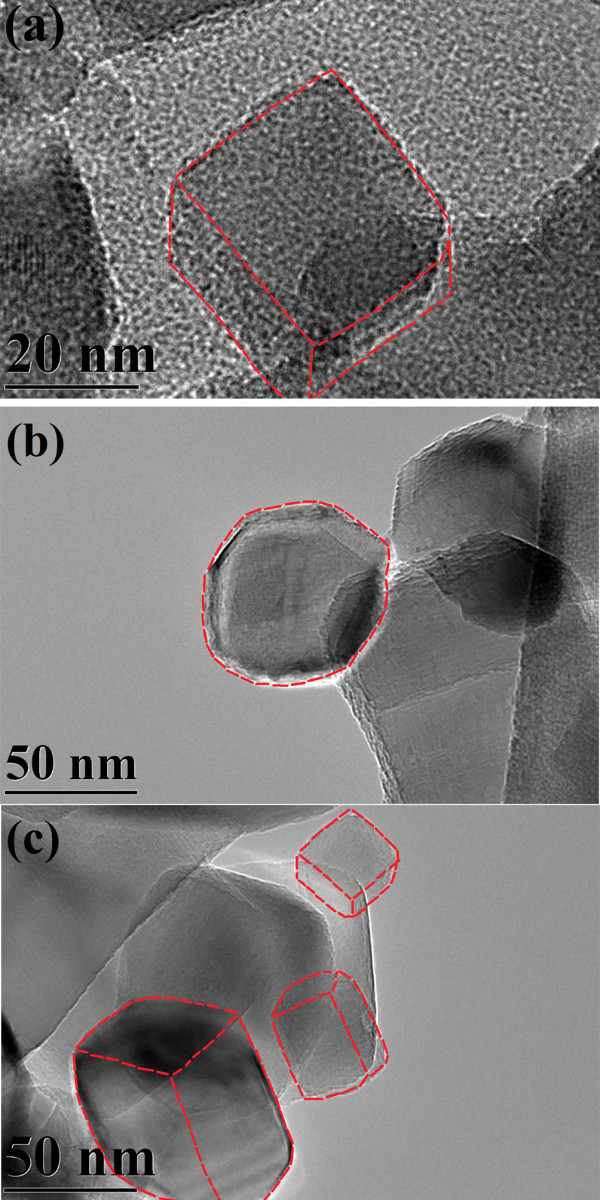
**TEM micrographs of single crystal for each shape of nanostructures. (a)** Cube, **(b)** sphere and **(c)** cube/cuboid.

**Figure 7:**
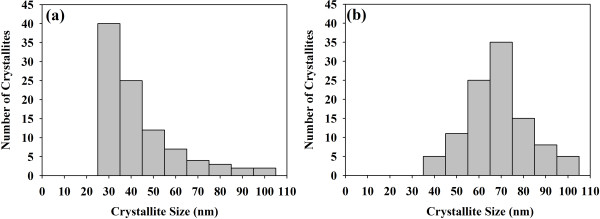
**Crystallite size distribution plots. (a)** MgO-OA and **(b)** MgO-TA.

Since the decomposition of the MgO-TA precursor starts at a lower temperature (380°C) compared to the MgO-OA precursor (420°C), the rate of MgO crystal growth will not be the same when identical thermal conditions are used on the precursors (950°C, 36 h). MgO-OA will have a slower rate of growth compared to MgO-TA resulting in smaller crystallites for MgO-OA. The two types of complexing agents seem to have quite different effects on the particle size of the MgO final products. It is remarkable that using these two types of complexing agents and annealing them at a relatively high temperature of 950°C with a long duration time of 36 h, the crystallite sizes of both samples are still very small as can be seen from the FESEM micrographs of Figure 4a,b for samples MgO-OA and MgO-TA, respectively. They show tiny crystallites of uniform size distribution. The shapes, however, are not clearly discernable due to the small size of the crystallites. This requires the higher resolution capability of a field emission TEM. The TEM micrographs in Figure 5a,b,c,d clearly show the shape and size of the MgO nanocrystals. The amorphous-like structure seen in the micrographs is actually the amorphous carbon of the lacy-type TEM grid and not an MgO feature. This is well known to electron microscopists involved in TEM work. The morphology of MgO-OA is cubic crystals while that of MgO-TA is of mixed cube, cuboid and spherical shapes. The high-magnification image shown in Figure 6a of the single crystal for MgO-OA is clearly evident of that of a cube while Figure 6b,c illustrates the shapes of sphere, cube and cuboid for the MgO-TA sample. The average crystallite size for MgO-OA is 30 nm which is smaller than MgO-TA with an average crystallite size of 68 nm. Figure 7 shows the crystallite size distribution plots for both samples. As can be seen, the size distribution characteristics for the two samples are different. For MgO-OA, there is a high frequency of crystallite size at the lower part of the size distribution plot while for MgO-TA, the size distribution is more of a normal type plot where the frequency is highest in the middle part of the plot at around 70 nm. Thus, not just the average crystallite size is different for the two samples but also the size distribution characteristics. These results demonstrate that the synthesis route employing tartaric acid has a faster growth rate than the one using oxalic acid. Oxalic acid and tartaric acid not only act as a complexing agent but also as a surfactant that inhibits crystal growth. These MgO nanostructures are believed to be very stable because they are prepared at a high temperature with a long annealing time. It is normal for MgO nanostructures not to have high stability because they are often annealed at lower temperatures for short periods of time [37-39].

**Figure 8:**
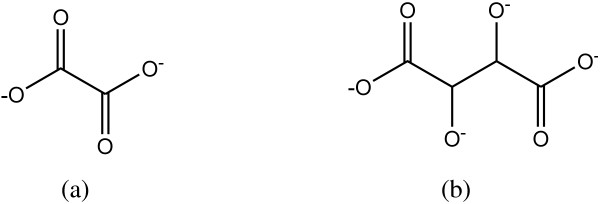
**The complexation sites available in the complexing agents. (a)** Oxalate and **(b)** tartrate.

As is well known, complexing agents play an important role in crystal formation by fixing the metal ions prior to the formation of the final product. We will, henceforth, propose an explanation for the effect of the complexing agents on the different crystallite sizes of the final products of MgO. Figure 8 shows that the complexation sites for tartaric acid are more numerous than those for oxalic acid. The oxalic acid, due to its smaller molecular structure with only two complexation sites, can fix less Mg^2+^ ions compared to the larger tartrate molecule. The tartrate molecule has more complexation sites and will be able to fix a larger number of Mg^2+^ ions, thus producing larger crystals.

**Figure 9:**
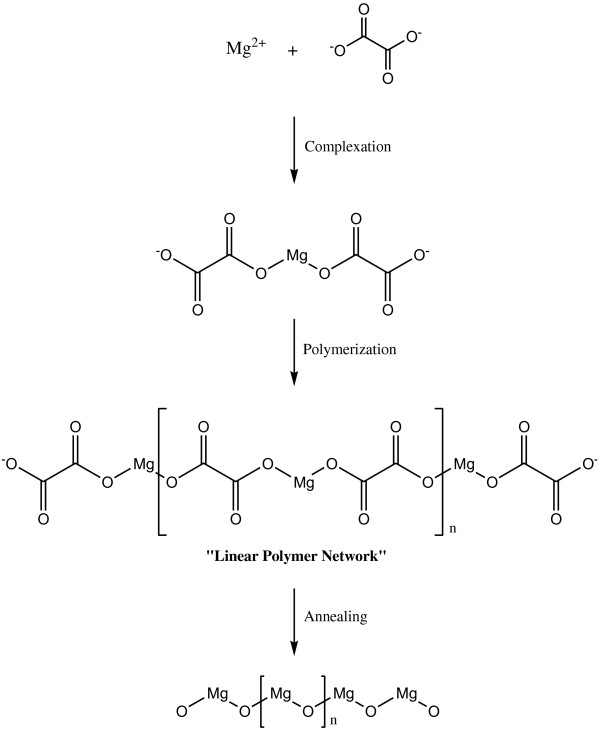
The growth mechanism for MgO-OA.

**Figure 10:**
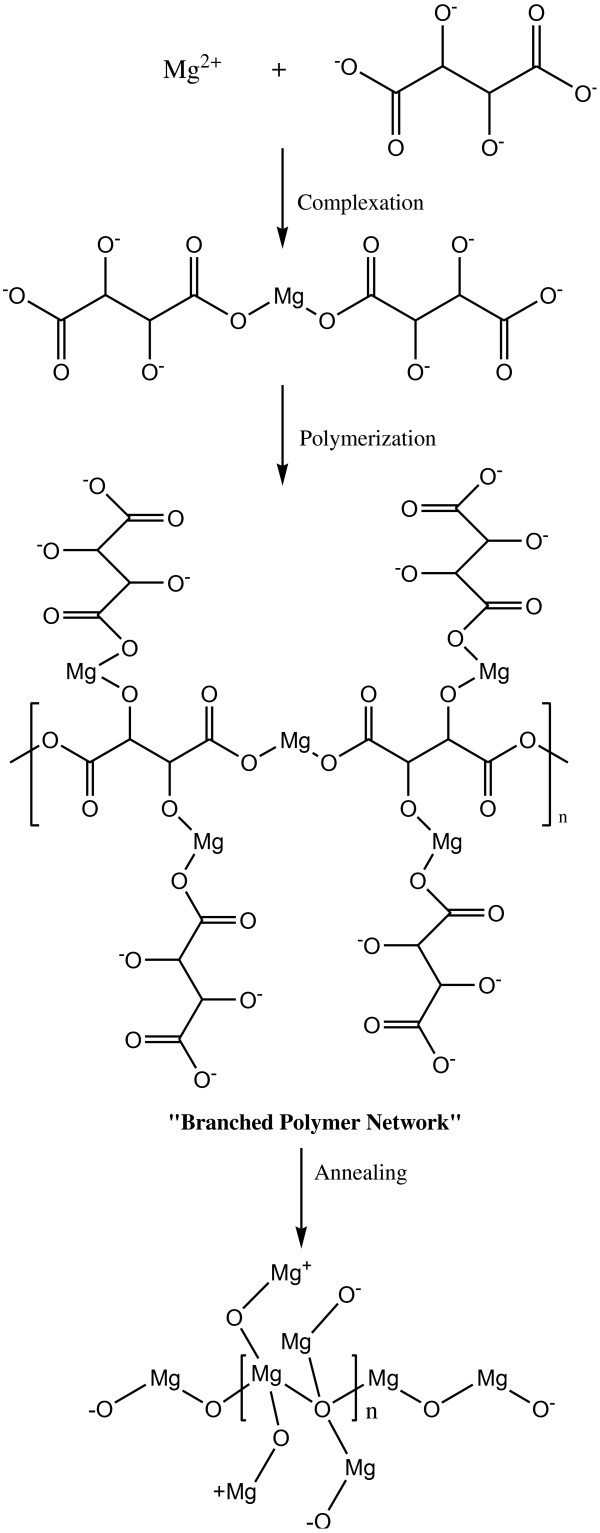
The growth mechanism for MgO-TA.

**Figure 11:**
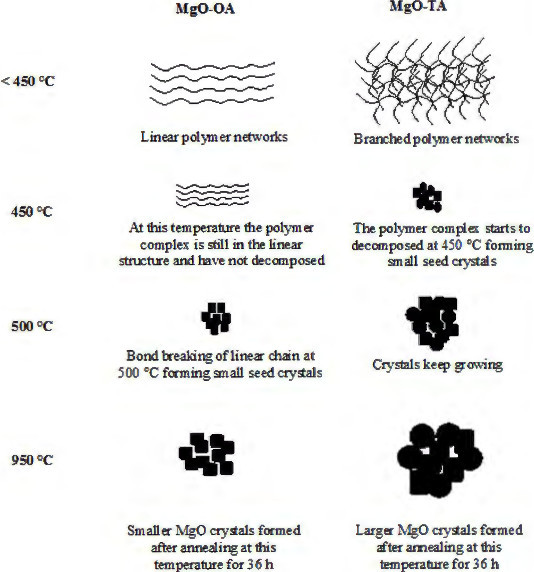
A schematic diagram for crystal growth of the MgO samples.

Figures 9 and 10 illustrate the growth mechanisms of the MgO nanostructures. Linear polymer networks are expected to be formed for oxalic acid during the sol-gel reaction due to the position of the two complexation sites being at the end of the polymer chain that can bind the Mg^2+^ ions forming the Mg-O ionic bonds as shown in Figure 9. For the tartaric acid complexing agent, the available four complexation sites at various positions for the attachments of the Mg^2+^ ions will result in branched polymer networks being formed as shown in Figure 10. The branched polymer networks that formed during the sol-gel reaction influence the crystallite growth. In the sol-gel route, the linear polymer networks can be packed close to one another to produce very dense macromolecules which decompose at a higher temperature. In contrast, the branched polymer networks form larger masses which are more unstable and can be decomposed at a lower temperature as is illustrated in Figure 11. This explanation agrees very well with the STA results of the MgO precursors. Therefore, at the same annealing condition (950°C, 36 h), the MgO-TA crystals start to nucleate earlier and have a faster growth rate compared to the MgO-OA crystals, which explains the mechanism of crystal growth and the effect of the structure of the complexing agents on the final size of the MgO nanocrystals.

## Conclusions

The use of oxalic acid and tartaric acid has been demonstrated to be very useful in producing thermally stable MgO nanostructures with a relatively uniform particle size. The growth mechanisms of the MgO nanostructures have been attributed to the very different molecular structures of the complexing agents which affected the crystal growth rate of MgO giving different crystallite sizes of the final products. The molecular structures and complexation site density play an important role in the fixing of the metal cation, Mg^2+^, and the formation of MgO nanoparticles. It is also clear that MgO-OA is able to produce nanocrystals not only of narrower size distribution but also of uniform morphology.

## Abbreviations

FESEM: field emission scanning electron microscope; OA: oxalic acid; STA: simultaneous thermogravimetric analysis; TA: tartaric acid; TEM: transmission electron microscope; XRD: X-ray diffraction.

## Competing interests

The authors declare that they have no competing interests.

## Authors’ contributions

MSM carried out the synthesis and characterization of the samples, analyzed the results and wrote a first draft of the manuscript. NK (Kamarulzaman) supervised the research and revised the manuscript. RR and NK (Kamarudin) helped in data acquisition of the samples using a high-resolution transmission electron microscope and some analysis. MAN and AMM contributed some ideas for the growth mechanisms of the samples. All authors read and approved the final manuscript.

## References

[B1] Sathyamoorthy R Mageshwari K Mali SS Priyadharshini S Patil PS Effect of organic capping agent on the photocatalytic activity of MgO nanoflakes obtained by thermal decomposition route Ceram Int 2013 9 323 330 10.1016/j.ceramint.2012.06.028

[B2] Yuan G Zheng J Lin C Chang X Jiang H Electrosynthesis and catalytic properties of magnesium oxide nanocrystals with porous structures Mater Chem Phys 2011 9 387 391 10.1016/j.matchemphys.2011.06.058

[B3] Nga NK Hong PTT Lam TD Huy TQ A facile synthesis of nanostructured magnesium oxide particles for enhanced adsorption performance in reactive blue 19 removal J Colloid Interface Sci 2013 9 210 216 23489606 10.1016/j.jcis.2013.02.018

[B4] Wu Z Xu C Chen H Wu Y Yu H Ye Y Gao F Mesoporous MgO nanosheets: 1,6-hexanediamin-assisted synthesis and their applications on electrochemical detection of toxic metal ions J Phys Chem Solids 2013 9 1032 1038 10.1016/j.jpcs.2013.02.029

[B5] Zhang K An Y Zhang L Dong Q Preparation of controlled nano-MgO and investigation of its bactericidal properties Chemosphere 2012 9 1414 1418 10.1016/j.chemosphere.2012.06.007 22771175

[B6] Umar A Rahman MM Hahn Y-B MgO polyhedral nanocages and nanocrystals based glucose biosensor Electrochem Commun 2009 9 1353 1357 10.1016/j.elecom.2009.04.033

[B7] Anderson PJ Horlock RF Thermal decomposition of magnesium hydroxide Trans Faraday Soc 1962 9 1993 2004

[B8] Green J Calcination of precipitated Mg(OH)_2_ to active MgO in the production of refractory and chemical grade MgO J Mater Sci 1983 9 637 651 10.1007/BF00745561

[B9] Kim MG Dahmen U Searcy AW Structural transformations in the decomposition of Mg(OH)_2_ and MgCO_3_ J Am Ceram Soc 1987 9 146 154 10.1111/j.1151-2916.1987.tb04949.x

[B10] Veldurthi S Shin C-H Joo O-S Jung K-D Synthesis of mesoporous MgO single crystals without templates Microporous Mesoporous Mater 2012 9 31 36

[B11] Zhao Z Dai H Du Y Deng J Zhang L Shi F Solvo- or hydrothermal fabrication and excellent carbon dioxide adsorption behaviors of magnesium oxides with multiple morphologies and porous structures Mater Chem Phys 2011 9 348 356 10.1016/j.matchemphys.2011.02.073

[B12] Li H Li M Wang X Wu X Liu F Yang B Synthesis and optical properties of single-crystal MgO nanobelts Mater Lett 2013 9 80 82

[B13] Hahn R Brunner JG Kunze J Schmuki P Virtanen S A novel approach for the formation of Mg(OH)_2_/MgO nanowhiskers on magnesium: rapid anodization in chloride containing solutions Electrochem Commun 2008 9 288 292 10.1016/j.elecom.2007.12.007

[B14] Alavi MA Morsali A Syntheses and characterization of Mg(OH)_2_ and MgO nanostructures by ultrasonic method Ultrason Sonochem 2010 9 441 446 10.1016/j.ultsonch.2009.08.013 19762266

[B15] Al-Gaashani R Radiman S Al-Douri Y Tabet N Daud AR Investigation of the optical properties of Mg(OH)_2_ and MgO nanostructures obtained by microwave-assisted methods J Alloys Compd 2012 9 71 76

[B16] Murugan R Ramamoorthy K Sundarrajan S Ramakrishna S Magnesium oxide nanotubes: synthesis, characterization and application as efficient recyclable catalyst for pyrazolyl 1,4-dihydropyridine derivatives Tetrahedron 2012 9 7196 7201 10.1016/j.tet.2012.06.017

[B17] Selvam NCS Kumar RT Kennedy LJ Vijaya JJ Comparative study of microwave and conventional methods for the preparation and optical properties of novel MgO-micro and nano-structures J Alloys Compd 2011 9 9809 9815 10.1016/j.jallcom.2011.08.032

[B18] Sun R-Q Sun L-B Chun Y Xu Q-H Wu H Synthesizing nanocrystal-assembled mesoporous magnesium oxide using cotton fibres as exotemplate Microporous Mesoporous Mater 2008 9 314 322 10.1016/j.micromeso.2007.08.006

[B19] Nusheh M Yoozbashizadeh H Askari M Kobatake H Fukuyama H Mechanically activated synthesis of single crystalline MgO nanostructures J Alloys Compd 2010 9 715 720 10.1016/j.jallcom.2010.07.049

[B20] Kim SW Kim KD Moon DJ Shape controlled synthesis of nanostructured magnesium oxide particles in supercritical carbon dioxide with ethanol cosolvent Mater Res Bull 2013 9 2817 2823 10.1016/j.materresbull.2013.04.019

[B21] Zhou J Yang S Yu J Facile fabrication of mesoporous MgO microspheres and their enhanced adsorption performance for phosphate from aqueous solutions Colloids Surf A Physicochem Eng Asp 2011 9 102 108 10.1016/j.colsurfa.2010.11.050

[B22] Sutradhar N Sinhamahapatra A Roy B Bajaj HC Mukhopadhyay I Panda AB Preparation of MgO nano-rods with strong catalytic activity via hydrated basic magnesium carbonates Mater Res Bull 2011 9 2163 2167 10.1016/j.materresbull.2011.02.024

[B23] Gao G Xiang L Emulsion-phase synthesis of honeycomb-like Mg_5_(OH)_2_(CO_3_)_4_.4H_2_O micro-spheres and subsequent decomposition to MgO J Alloys Compd 2010 9 242 246 10.1016/j.jallcom.2010.01.138

[B24] Bartley JK Xu C Lloyd R Enache DI Knight DW Hutchings GJ Simple method to synthesize high surface area magnesium oxide and its use as a heterogeneous base catalyst Appl Catal B 2012 9 31 38

[B25] Ganguly A Trinh P Ramanujachary KV Ahmad T Mugweru A Ganguli AK Reverse micellar based synthesis of ultrafine MgO nanoparticles (8-10 nm): characterization and catalytic properties J Colloid Interface Sci 2011 9 137 142 10.1016/j.jcis.2010.09.041 20934186

[B26] Lopez T Garcia-Cruz I Gomez R Synthesis of magnesium oxide by the sol-gel method: effect of the pH on the surface hydroxylation J Catal 1991 9 75 85 10.1016/0021-9517(91)90210-U

[B27] Bokhimi X Morales A Lopez T Gomez R Crystalline structure of MgO prepared by the sol-gel technique with different hydrolysis catalysts J Solid State Chem 1995 9 411 415 10.1006/jssc.1995.1152

[B28] Wang JA Novaro O Bokhimi X Lopez T Gomez R Navarrete J Llanos ME Lopez-Salinas E Characterizations of the thermal decomposition of brucite prepared by sol-gel technique for synthesis of nanocrystalline MgO Mater Lett 1998 9 317 323 10.1016/S0167-577X(97)00273-5

[B29] Kumar A Kumar J Defect and adsorbate induced infrared modes in sol-gel derived magnesium oxide nano-crystallites Solid State Commun 2008 9 405 408 10.1016/j.ssc.2008.06.014

[B30] Kumar A Kumar J On the synthesis and optical absorption studies of nano-size magnesium oxide powder J Phys Chem Solids 2008 9 2764 2772 10.1016/j.jpcs.2008.06.143

[B31] Kumar A Thota S Varma S Kumar J Sol-gel synthesis of highly luminescent magnesium oxide nanocrytallites J Lumin 2011 9 640 648 10.1016/j.jlumin.2010.11.008

[B32] Sharma M Jeevanandam P Synthesis of magnesium oxide particles with stacks of plates morphology J Alloys Compd 2011 9 7881 7885 10.1016/j.jallcom.2011.04.151

[B33] Putanov P Kis E Boskovic G Effects of the method of preparation of MgC_2_O_4_ as a support precursor on the properties of iron/magnesium oxide catalysts Appl Catal 1991 9 17 26 10.1016/0166-9834(91)85109-9

[B34] Yan L Zhuang J Sun X Deng Z Li Y Formation of rod-like Mg(OH)_2_ nanocrystallites under hydrothermal conditions and the conversion to MgO nanorods by thermal dehydration Mater Chem Phys 2002 9 119 122 10.1016/S0254-0584(01)00509-0

[B35] Jung HS Lee J-K Kim JY Hong KS Synthesis of nano-sized MgO particle and thin film from diethanolamine-stabilized magnesium-methoxide J Solid State Chem 2003 9 278 283 10.1016/S0022-4596(03)00280-9

[B36] Trionfetti C Babich IV Seshan K Lefferts L Formation of high surface area Li/MgO: efficient catalyst for the oxidative dehydrogenation/cracking of propane Appl Catal A Gen 2006 9 105 113

[B37] Venkatesha TG Nayaka YA Chethana BK Adsorption of Ponceau S from aqueous solution by MgO nanoparticles Appl Surf Sci 2013 9 620 627

[B38] Mehta M Mukhopadhyay M Christian R Mistry N Synthesis and characterization of MgO nanocrystals using strong and weak bases Powder Technol 2012 9 213 221

[B39] Bhatte KD Sawant DN Deshmukh KM Bhanage BM Additive free microwave assisted synthesis of nanocrystalline Mg(OH)_2_ and MgO Particuol 2012 9 384 387 10.1016/j.partic.2011.05.004

